# Hydrogen sulfide (H_2_S) emission control by aerobic sulfate reduction in landfill

**DOI:** 10.1038/srep38103

**Published:** 2016-12-02

**Authors:** Yuyang Long, Yuan Fang, Dongsheng Shen, Huajun Feng, Ting Chen

**Affiliations:** 1 Zhejiang Provincial Key Laboratory of Solid Waste Treatment and Recycling, School of Environmental Science and Engineering, Zhejiang Gongshang University, Hangzhou, 310018, China

## Abstract

H_2_S emissions from landfill sites resulting from sulfate reduction has become a serious human health and ecological safety issue. This study investigated H_2_S emission behavior and sulfate metabolism occurring in simulated landfills under different operating conditions. Under aerobic conditions, great attenuation of the original sulfate content (from around 6000 mg kg^−1^ dropped to below 800 mg kg^−1^) with corresponding accumulation of sulfides and elemental sulfur were observed, indicating that sulfate reduction processes were intense under such conditions. Analysis of the bacterial community in these landfills showed great abundance (1.10%) and diversity of sulfur reducing types, confirming their active involvement in this process. In particular, the total abundance of sulfate-reducing bacteria increased nearly 30 times under aerobic conditions, leading to the transformation of sulfate to sulfide and other reduced sulfur species. Although exposure to air promoted the accumulation of sulfide, it did not lead to an increase in H_2_S release in these landfills.

Odor emission from landfill sites related to H_2_S, which has an extremely low odor threshold (around 0.5 ppb)^1^ and high toxicity, has become an environmental problem, linked with wide-scale public complaint. H_2_S can cause eye irritation at concentrations as low as 50–100 ppm, and concentrations of 300–500 ppm may result in severe poisoning, leading to unconsciousness and death^1^. It can seriously endanger human health and ecology safety. The formation of H_2_S from landfill sites mainly results from anaerobic biological conversion of sulfate by sulfate-reducing bacteria (SRB)^2^,^3^, considered to be obligate anaerobes^4^. However, some studies have shown that this reduction process can also occur in the presence of O_2_. Canfield *et al*.^5^ first revealed that sulfate reduction occurred consistently within the well-oxygenated photosynthetic zone of bacterial mats; however it is uncertain whether sulfate reduction also occurs in the aerobic zone of more typical, nonphotosynthetic clastic marine sediments. Wang *et al*.^6^ metabolically engineered a novel aerobic sulfate reduction pathway, redirecting the assimilatory sulfate reduction pathway to overproduce cysteine to secrete sulfides. Sigalevich *et al*.^7^ found that sulfate reduction could be detected during co-culture of SRB (*Desulfovibrio oxyclinae*) together with a facultative aerobic heterotroph (*Marinobacter* sp.) under anaerobic and aerobic conditions (with 0 to 20% O_2_). These observations challenge the conventional view that sulfate reduction is a strictly anaerobic process. In recent years, limited oxygen supply to anaerobic wastewater treatment systems had been demonstrated as an effective strategy to improve elemental sulfur recovery, coupling sulfate reduction and sulfide oxidation^8^,^9^,^10^. Yu *et al*.^8^ supposed that this phenomenon might be due to the significant influence of dissolved oxygen on the microbial functional communities, but to date, the related mechanisms are still unclear. Celis-García *et al*.^11^ successfully transformed sulfate into elemental sulfur under low aeration rate of 2.3 L d^−1^ in a down-flow fluidized bed reactor. Sahinkaya *et al*.^10^ also revealed that elemental sulfur recovery could be controlled by manipulating the sulfide loading and oxygen pressure.

Landfill is a typical anaerobic environment. However, with the introduction of water and air through leachate recirculation and semi-aerobic landfilling, more of the landfill is exposed to air, which is beneficial for stabilization. In such scenarios, release of reduced substances like H_2_S change the surrounding environment. A number of studies have focused on H_2_S emission behavior related to air exposure. Zhang *et al*.^12^ demonstrated effects of air venting and moisture variation on H_2_S production in aged-construction and demolition debris landfills. The results showed that air venting immediately suppressed biological H_2_S production, while moisture addition retained more stable H_2_S levels over the long term. In our previous studies, H_2_S emission from refuse exposed to air was investigated in laboratory batch test experiments^13^. The results showed that large amounts of H_2_S can be released from aged refuse, when it is re-excavated and exposed to air, especially within the first few hours. It was hypothesized that this involved aerobic sulfate reduction. Therefore, the experimental scale was enlarged from a laboratory microcosm to simulated landfill reactor to determine the processes involved in this conversion of sulfur compounds. It aimed to identify aerobic sulfate reduction processes occurring in such landfills.

## Methods

### Experimental set-up

Six simulated bioreactor landfill sets, constructed with watertight polypropylene, were established in the study. Anaerobic landfill (AL) and anaerobic landfill with leachate recirculation (RAL) were used as control sets. Two experiments involved anaerobic landfills being switched to semi-aerobic mode after day 175, using direct discharge (ASL) and leachate recirculation (RASL); while the other two experiments were run as semi-aerobic landfill (SL) and semi-aerobic landfill with leachate recirculation (RSL) (see Supplementary Fig. S1). Each bioreactor had an internal diameter of 0.5 m and a height of 2.0 m. They comprised a 100-mm-thick layer of headspace, a 1600-mm-thick layer of landfill, and a 300-mm-thick layer for leachate collection. Each bioreactor was packed with freshly collected municipal solid waste (MSW) from the transport station of Hangzhou (Zhejiang, China), with a wet density of 880 kg m^−3^. The characteristics of the MSW used in this experimental were (by wet weight, w/w): food and fruit waste, 66.8%; plastic, 3.2%; paper, 9.2%; dust, 1.8%; glass, 0.4%; cellulose textile, 0.5%; metal, 0.2%; timber, 1.6%; residue, 16.5%. The moisture content of the MSW was 68%. The MSW was loaded in 1450-mm layers and compacted using a shovel and sledgehammer. A 100-mm-thick layer of gravel and a 50-mm-thick layer of sand were placed at the bottom and top of the MSW layers, respectively. The MSW layer was divided into three layers: shallow, middle and deep, with each layer having three sampling ports separated by an angle of 120°. There were two inlet/outlet ports in the top lid for gas removal or leachate recycling (only for the RAL, RASL and RSL cases) and one port at the bottom of the landfill for leachate drainage and sampling. In addition, the four aerobic experiments had three inlets at the bottom of each landfill, and a central vent pipe with a diameter of 5 cm in the middle of the landfill. These three inlets were also distributed at 120° angles. The semi-aerobic system achieved through a convection process. Before day 175, the inlets and central vent pipes in both the ASL and RASL were closed to maintain anaerobic conditions.

### Sample collection

Refuse was sampled periodically from all three layers from the sampling ports around the side of each simulated landfill. Approximately 100 g refuse samples were collected from each refuse sampling port in a given layer, and mixed to form a lager sample (~300 g), of which 20 g were stored at −80 °C for bacterial community analysis. Leachate was collected from the leachate outlet ports (~100 mL). To maintain equilibrium of leachate volume in the landfills with leachate recirculation, the same volume of tap water (~100 mL) was added back into the leachate after sampling. Gas samples were also monitored periodically from the gas outlet port in the top lid, as well as in the nine ports around the landfills. Because of landfill settlement caused by rapid degradation of the refuse, samples in the shallow layers of the ASL and RASL could not be collected after day 269, while samples in the shallow layers of the SL and RSL could not be collected after day 191.

### Chemical analysis

Refuse samples were analyzed for pH, moisture content, dissolved organic carbon, as well as sulfate (SO_4_^2−^), sulfide (H_2_S, HS^−^, S^2−^), ferrous (Fe^2+^), nitrate (NO_3_^−^) and nitrite (NO_2_^−^) contents after samples were passed through a 0.22-μm filter^14^.

Leachate samples were analyzed for volume, pH, chemical oxygen demand, dissolved organic carbon, volatile fatty acids, sulfide and sulfate contents^14^. Gas samples were analyzed specifically for H_2_S^14^ and CO_2_^15^.

All analyses were carried out in triplicate to ensure the validity of our results. Results of all chemical analyses were calculated on a dry-weight basis.

### Bacterial community analysis

Refuse samples from three key time points (0 d, 191 d and 269 d) were selected for bacterial community analysis. These were labeled to analyze and map (see Supplementary Table S1). The genomic DNA of each sample was extracted using an extraction kit (DR4011; Bioteke Corporation, Beijing, China) according to the manufacturer’s instructions. The bacterial 16S rRNA gene of the extracted DNA was amplified using the primer pair 338F (5′-ACTCCTACGGGAGGCAGCAG-3′) and 806 R (5′-GGACTACATCGACGGGTATTCTAAT-3′)^16^. The bacterial community was investigated using Illumina high-throughput sequencing carried out by Majorbio Bio-Pharm Technology Co., Ltd (Shanghai, China)^17^. Raw pyrosequencing data obtained were deposited to the NCBI Sequence Read Archive (SRA, http://www.ncbi.nlm.nih.gov/Traces/sra). SRA accession number was SAMN04505227 for the bacterial raw pyrosequencing data. Sequences were clustered into operational taxonomic units by setting a 0.03 distance limit (equivalent to 97% similarity) using the Usearch program. Sequences were phylogenetically assigned to taxonomic classifications using an RDP classifier and were allocated to different levels^17^.

### Statistical analysis

Statistical analyses were conducted using the R Statistics Program. Figures were drawn using Origin 8.5 and Surfer 10. All figures have symbols with bars to represent standard deviation. A relative abundance heatmap was created using genus-level sulfur-metabolizing bacterial annotations. Prior to statistical analysis, relative abundance was log(x + 1)-transformed to obtain a normal distribution. Multivariate analysis was conducted using canonical correspondence analysis (CCA) of genus-level sulfur-metabolizing bacterial taxa and refuse property data. Calculations were performed using the CCA function from the vegan package in the R Statistics Program, with refuse property data as environmental parameters.

### Landfill sulfur mass balance

(1) Total sulfur input in the initial stage: the total sulfur concentration in the original refuse was 1560 mg kg^−1^, and the total weight of refuse loaded in each set of simulated landfill was 250 kg. So the initial total sulfur content in each simulated landfill was 390 g.

(2) Sulfur output during the landfilling: during the operating process of simulated landfills, ignoring the reduce amount of refuse caused by gas release, and then the reduce amount of refuse was mainly caused by leachate discharge and sampling. The sulfur content of each form in simulated landfills without leachate recirculation could be calculated by Eq. 1, while in simulated landfills with leachate recirculation, it could be calculated by Eq. 2.


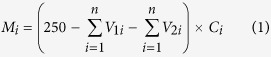



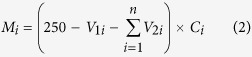


In which *M*_*i*_ is the sulfur content of certain form in simulated landfills on day *i; V*_*1i*_ is the leachate amount on day *i; V*_*2i*_ is the refuse samples amount collected on day *i; C*_*i*_ is the sulfur concentration of certain form in middle layer of simulated landfills on day *i*.

(3) Other forms of sulfur mainly include sulfur-containing organic compound and sulfur-containing inorganic compound except for sulfide, elemental sulfur, thiosulfate and sulfate.

## Results

### Sulfate attenuation in landfills

**Figure 1:**
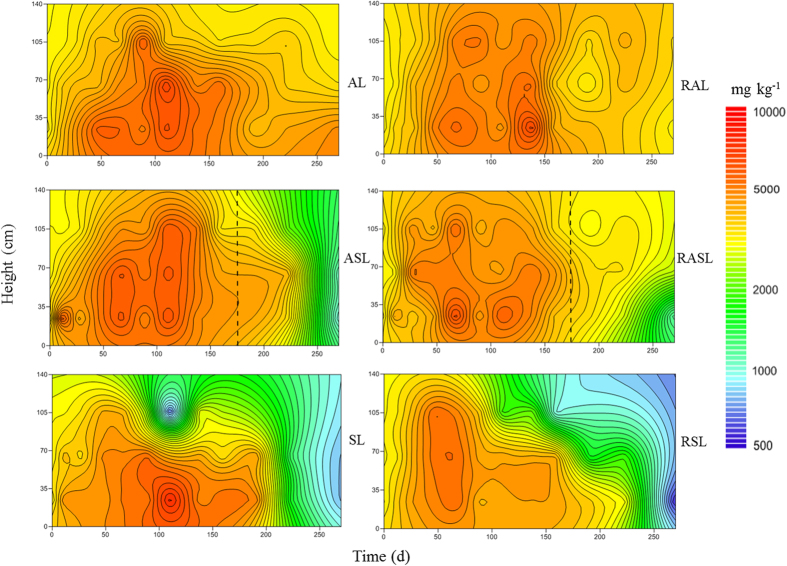
The response contour plot of sulfate concentration in simulated landfill. (AL: anaerobic landfill; RAL: anaerobic landfill with leachate recirculation; ASL: anaerobic landfills being switched to semi-aerobic mode after day 175; RASL: anaerobic landfills being switched to semi-aerobic mode after day 175 with leachate recirculation; SL: semi-aerobic landfill; RSL: semi-aerobic landfill with leachate recirculation. The unit of sulfate content is mg SO_4_^2−^ kg^−1^).

Sulfate reduction is the main source of H_2_S production in landfill sites. As shown in Fig. 1, the sulfate contents in all six simulated landfills underwent different growth trends, before attenuating. The initial sulfate content of the raw MSW before landfilling was 3198 mg kg^−1^. With the rapid degradation of the refuse, the sulfate content increased sharply because of hydrolysis. The highest sulfate content in all simulated landfills was around 6000 mg kg^−1^. As time went by, the sulfate content in anaerobic landfills (AL and RAL) decreased slightly, with the lowest content still more than 3000 mg kg^−1^. While ASL and RASL showed an obvious decline in sulfate content after being switched to semi-aerobic mode, and the lowest contents were 1015 mg kg^−1^ and 1172 mg kg^−1^, respectively. In contrast, the sulfate contents in the SL and RSL, both operating under semi-aerobic conditions at all times, decreased significantly. In particular, the sulfate content in the shallow layer having good contact with air, dropped to below 800 mg kg^−1^ by day 269. The results showed that being exposed to air promoted reduction of sulfate. However, this was not related to leachate washing out (see Supplementary Fig. S2), but to biological transformation.

### Sulfide and elemental sulfur accumulation in landfills

**Figure 2:**
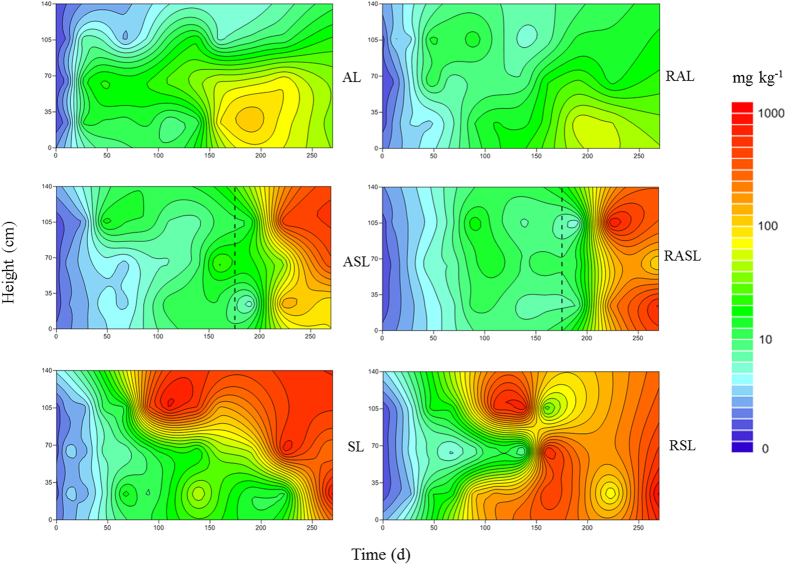
The response contour plot of sulfide concentration in simulated landfill. (AL: anaerobic landfill; RAL: anaerobic landfill with leachate recirculation; ASL: anaerobic landfills being switched to semi-aerobic mode after day 175; RASL: anaerobic landfills being switched to semi-aerobic mode after day 175 with leachate recirculation; SL: semi-aerobic landfill; RSL: semi-aerobic landfill with leachate recirculation. The unit of sulfide content is mg sulfide kg^−1^).

Sulfide is the reduction product of high-valence sulfur, and it is a potential source of H_2_S^18^. As shown in Fig. 2, different levels of sulfide accumulation occurred in the six simulated landfills during stabilization. Under anaerobic conditions, our AL and RAL showed initial increases in sulfide content, but changes were not marked, and their highest contents were 118 mg kg^−1^ and 59 mg kg^−1^ on day 191, respectively. In contrast, our SL and RSL did not show any obvious accumulation of sulfide before day 89, followed by a huge increase that reached peak values of 851 mg kg^−1^ and 1046 mg kg^−1^ on day 269. This behavior is supported by ASL and RASL experiments. Before being switched to semi-aerobic mode, the sulfide contents remained below 80 mg kg^−1^. When exposed to aerobic conditions, their sulfide contents increased rapidly, with highest contents of 467 mg kg^−1^ and 815 mg kg^−1^ on day 223, respectively. Coincident with the cumulative release of sulfide in the leachate (see Supplementary Fig. S3), their sulfide contents clearly increased on exposure to air.

Elemental sulfur has strong redox properties and is an important intermediate in any sulfate conversion process^19^. However elemental sulfur (see Supplementary Table S2) was not detected in the AL and RAL, but was detected in each layer of refuse in the SL and RSL, with highest concentrations of 1568 mg kg^−1^ and 2099 mg kg^−1^, respectively. Similarly, elemental sulfur was only detected in the ASL and RASL after exposure to air; at this time, elemental sulfur concentration increased rapidly, especially in the shallow and deep layers of refuse, from less than 100 mg kg^−1^ to more than 1000 mg kg^−1^. Thus, the results showed that a limited oxygen supply to anaerobic systems could improve elemental sulfur recovery. Moreover, in this study, thiosulfate was only detected in the SL and RSL (see Supplementary Table S3).

### Aerobic sulfate-reducing behavior

**Figure 3:**
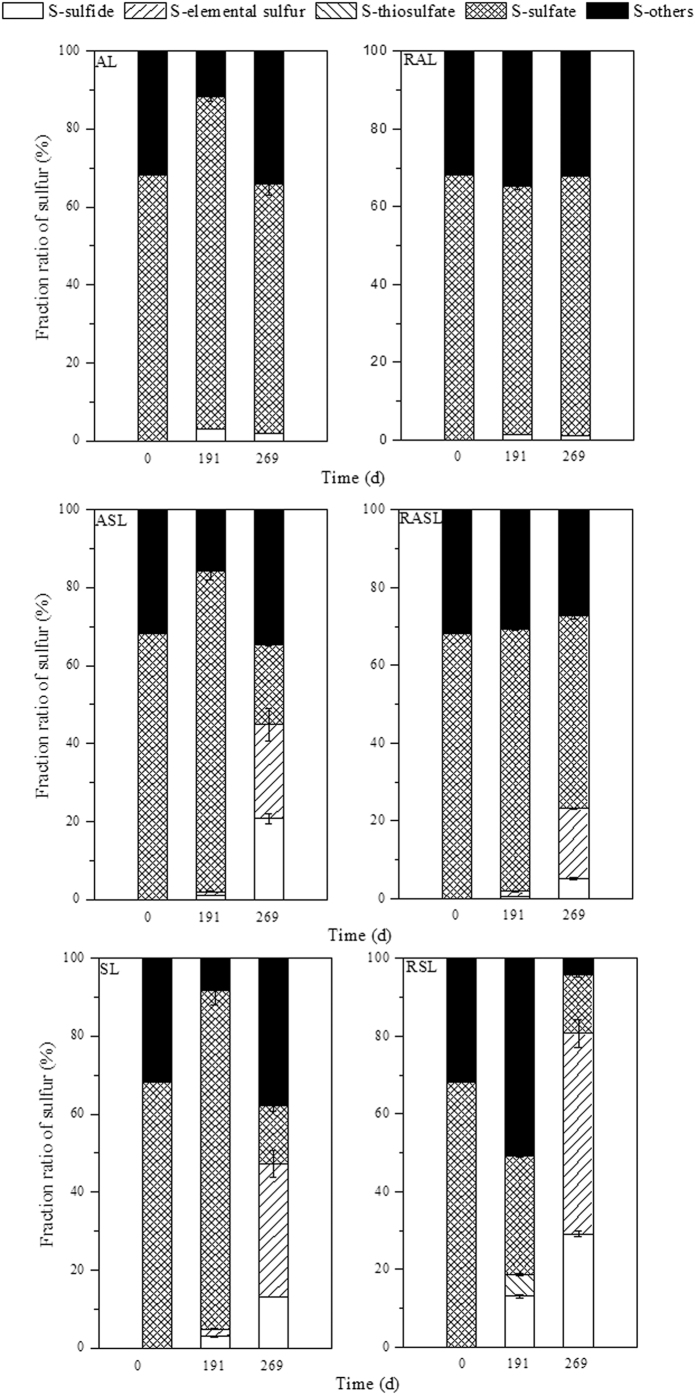
Sulfur fate in the refuse layers during the stabilization of the simulated landfills. (AL: anaerobic landfill; RAL: anaerobic landfill with leachate recirculation; ASL: anaerobic landfills being switched to semi-aerobic mode after day 175; RASL: anaerobic landfills being switched to semi-aerobic mode after day 175 with leachate recirculation; SL: semi-aerobic landfill; RSL: semi-aerobic landfill with leachate recirculation. S-sulfide, S-elemental sulfur, S-thiosulfate, S-sulfate and S-others represent the sulfur fraction ratio of sulfide, elemental sulfur, thiosulfate, sulfate and other forms of sulfur, respectively).

Sulfur mass balance calculations (see Fig. 3) showed that more than 60% of the sulfur existed as sulfate under anaerobic conditions in this study. However, the introduction of air promoted sulfate reduction to low valence sulfur, increasing both sulfide and elemental sulfur contents.

**Figure 4:**
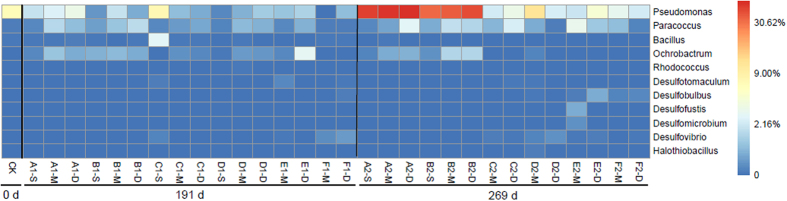
Percent relative abundance heatmap of microbial communities (genus level). (The nomenclature of the samples is shown in Supplementary Table S1 A~F represent the samples taken from AL, RAL, ASL, RASL, SL, RSL; S, M, D represent shallow, middle, deep layer of each simulated landfill; CK represents the original refuse sample; A1-S~F1-D represent the samples taken at day 191, andA2-S~F2-D represent the samples taken at day 269).

Typical sulfur-metabolizing bacteria genera were detected in all six simulated landfills (see Fig. 4 and Supplementary Table S4), including *Desulfobulbus, Desulfofustis, Desulfomicrobium, Desulfovibrio, Desulfotomaculum, Bacillus, Halothiobacillus, Ochrobactrum, Paracoccus, Pseudomonas* and *Rhodococcus*. Among them, the first five genera are affiliated with SRB. *Pseudomonas* was dominant in the initial refuse sample (6.76%), where the total abundance of SRB was less than 0.01%. On day 191, *Pseudomonas, Paracoccus* and *Ochrobactrum* were abundant in all simulated landfill samples. The total abundance of SRB under semi-aerobic conditions reached a peak of 0.62%, approximately 20 times and 3 times that observed under anaerobic conditions and semi-aerobic switched form anaerobic conditions, respectively. On day 269, the AL and RAL were still dominated by these three genera, and there were not obvious changes to the total SRB abundance. While in semi-aerobic switched from anaerobic landfills and semi-aerobic landfills, the total SRB abundance continued to increase, with abundances reaching 0.38% and 1.10%, or about 10 times and 30 times that under anaerobic conditions. Clearly, during the stabilization process, the sulfur-metabolizing bacterial community diversity improved. On exposure to air, the relative abundance of SRB (e.g., *Desulfobulbus*) increased nearly 30 times. Using canonical correspondence analysis (see Supplementary Fig. S4), it can be showed that the environmental changes caused by our different operating conditions had a significant influence on the composition and distribution of the sulfur-metabolizing bacterial community. The dominant genera were mainly SRB (such as *Desulfotomaculum* and *Desulfovibrio*) under semi-aerobic conditions, while they were sulfur-oxidizing bacteria (such as *Rhodococcus, Paracoccus, Ochrobactrum*, and *Pseudomonas*) under anaerobic conditions.

### Environmental implications

**Figure 5:**
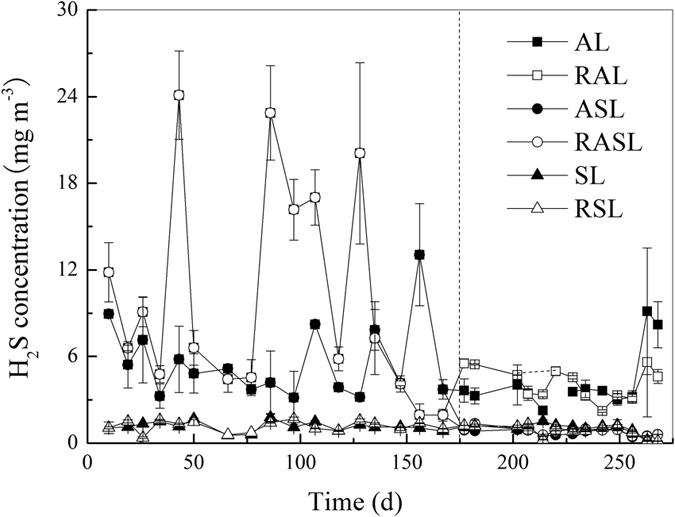
Release behavior of H_2_S from the cover layer of simulated landfills. (AL: anaerobic landfill; RAL: anaerobic landfill with leachate recirculation; ASL: anaerobic landfills being switched to semi-aerobic mode after day 175; RASL: anaerobic landfills being switched to semi-aerobic mode after day 175 with leachate recirculation; SL: semi-aerobic landfill; RSL: semi-aerobic landfill with leachate recirculation. The unit of H_2_S concentration is mg H_2_S m^−3^).

Theoretically, SRB can use sulfate as an electron acceptor to produce sulfide, often released as H_2_S^19^. In this study, H_2_S emission concentrations were significantly lower under semi-aerobic conditions than under anaerobic ones (see Fig. 5). The H_2_S concentration in the SL and RSL remained at a low level of no more than 2.0 mg m^−3^. Before day 175, the other four simulated landfills were all under anaerobic conditions, with H_2_S concentrations much higher than for the SL and RSL. After the ASL and RASL were switched to semi-aerobic operating mode, H_2_S concentrations decreased to less than 1.0 and 1.3 mg m^−3^ for the remainder of the experiment, respectively. Although the H_2_S concentrations in the AL and RAL decreased over this period, they were still higher than for all four semi-aerobic landfills.

**Figure 6:**
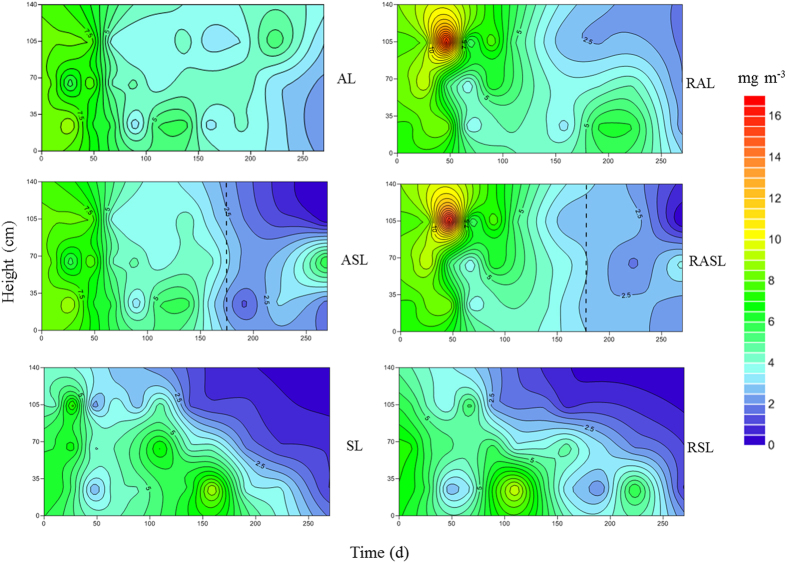
The response contour plot of H_2_S concentration in simulated landfill. (AL: anaerobic landfill; RAL: anaerobic landfill with leachate recirculation; ASL: anaerobic landfills being switched to semi-aerobic mode after day 175; RASL: anaerobic landfills being switched to semi-aerobic mode after day 175 with leachate recirculation; SL: semi-aerobic landfill; RSL: semi-aerobic landfill with leachate recirculation. The unit of H_2_S concentration is mg H_2_S m^−3^).

Inside landfills, H_2_S emissions should experience a long migration process. However, H_2_S concentration in our six simulated landfills generally had a downward trend (see Fig. 6). Lower H_2_S concentration was observed in SL and RSL experiments than in any other landfills, where H_2_S concentration in the shallow layer was lower than in middle and deep layers. Before day 175, H_2_S concentrations in the landfills operating under anaerobic conditions were significantly higher than in SL and RSL experiments, reaching 16.4 mg m^−3^. After transformation from anaerobic to semi-aerobic conditions, H_2_S concentrations substantially decreased to less than 2.0 mg m^−3^, but were still higher than the lowest H_2_S concentrations in the SL and RSL experiments (1.0 mg m^−3^). Late in our experiments, a slight decline in H_2_S concentrations was observed in the AL and RAL landfills, although these levels were still higher than for all four other simulated landfills. The results showed that H_2_S emission behavior from each layer was consistent with its release characteristics from the cover layer.

## Discussion

During the stabilization process, sulfate is converted into other sulfur forms by anaerobes (e.g., SRB) or assimilated to form organic containing sulfur (OCS)^20^. However, it was unexpected that the sulfate content in our anaerobic landfills (AL and RAL) did not diminish. On the contrary, aerobic conditions facilitated the transformation of sulfur-containing compounds in refuse, causing reduction of sulfate. On exposure to air, the relative abundance of SRB (e.g., *Desulfobulbus*) increased nearly 30 times, causing intense sulfate reduction in these semi-aerobic landfills. This eventually led to the significant accumulation of sulfide and elemental sulfur at these sites. It has been demonstrated that recovery of elemental sulfur is effectively controlled by the amount of oxygen in bioreactors, coupling sulfate reduction and sulfide oxidation^8^,^9^,^10^. Some microorganisms, such as *Desulfovibrio propionicus*, have been shown to be able to grow in the presence of O_2_, oxidizing sulfide and elemental sulfur to form sulfate or forming thiosulfate from elemental sulfur^21^. Such results indicate that sulfate reduction processes may be more intense under aerobic conditions, resulting in greater attenuation of sulfate content and accumulation of sulfide and elemental sulfur.

**Figure 7:**
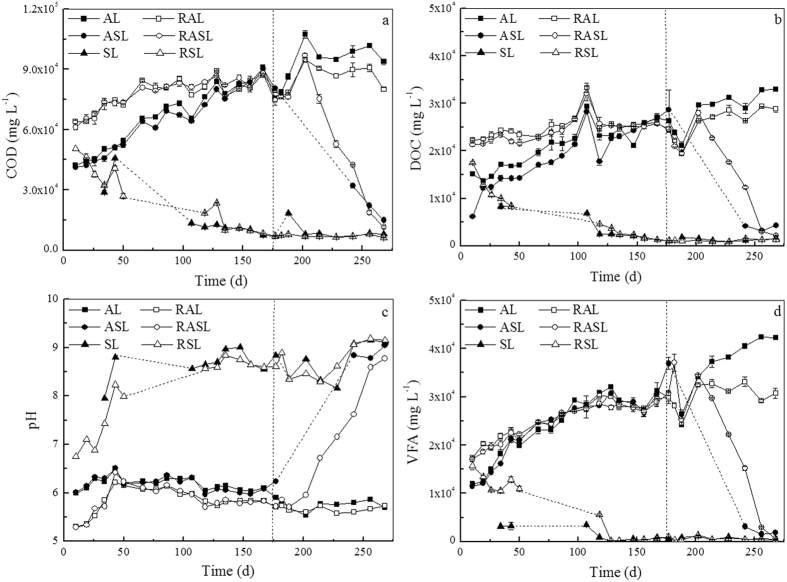
Changes of COD (**a**) DOC (**b**) pH (**c**) and VFA (**d**) in leachate from simulated landfills (AL: anaerobic landfill; RAL: anaerobic landfill with leachate recirculation; ASL: anaerobic landfills being switched to semi-aerobic mode after day 175; RASL: anaerobic landfills being switched to semi-aerobic mode after day 175 with leachate recirculation; SL: semi-aerobic landfill; RSL: semi-aerobic landfill with leachate recirculation).

Air exposure and leachate recirculation appear to accelerate the stabilization process in our study (see Fig. 7), resulting in fast degradation of organic substrates in the landfills. In addition, these conditions inhibited accumulation of organic acid, and caused pH to increase. Once H_2_S is generated in the biofilm, it diffuses into water within the MSW, where it dissociates into various species, depending on pH, as described by Lahav *et al*.^18^ and outlined in Eqs (3) and (4).


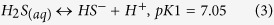



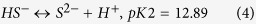


Thus, the emission of H_2_S is a physicochemical process that involves both water and air, and is pH sensitive. Only H_2_S_(aq)_ can transfer across the air-water interface, allowing H_2_S(aq) to be emitted as gas from MSW, as shown in Eq. (5)^19^.


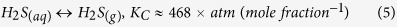


Between pH 5–9, the fraction of available H_2_S decreases as pH increases because of its dissociation into HS^−^22. Higher pH in the SL and RSL led to less H_2_S available to be transferred from the MSW into the atmosphere, indicating that introduction of air into landfills could reduce the external release of H_2_S, reducing the risk of “stench”.

Although being exposed to the air promoted sulfate reduction, causing obvious sulfide accumulation in our landfills, this accumulation did not lead to an increase in H_2_S release in landfills.

## Conclusions

During the landfill stabilization process, the sulfur-metabolizing bacterial community clearly diversified. In particular, the total abundance of SRB increased nearly 30 times on exposure to air, causing intense sulfate reduction and eventually leading to a significant accumulation of sulfide and elemental sulfur in our landfills. Although H_2_S emission potential in landfills under aerobic conditions was not improved; the reason for this reservoir of sulfide, not resulting in H_2_S emission is yet to be determined.

## Additional Information

**How to cite this article**: Long, Y. *et al*. Hydrogen sulfide (H_2_S) emission control by aerobic sulfate reduction in landfill. *Sci. Rep.* **6**, 38103; doi: 10.1038/srep38103 (2016).

**Publisher's note:** Springer Nature remains neutral with regard to jurisdictional claims in published maps and institutional affiliations.

## Supplementary Material

Supplementary Information

## References

[b1] Firer D., Friedler E. & Lahav O. Control of sulfide in sewer systems by dosage of iron salts: Comparison between theoretical and experimental results, and practical implications. Sci. Total Environ. 392, 145–156 (2008). 18158171 10.1016/j.scitotenv.2007.11.008

[b2] Ding Y. . Characterization and control of odorous gases at a landfill site: A case study in Hangzhou, China. Waste Manage. 32, 317–326 (2012). 10.1016/j.wasman.2011.07.016 22137772

[b3] Panza D. & Belgiorno V. Hydrogen sulphide removal from landfill gas. Process Saf. Environ. 88, 420–424 (2010).

[b4] Barton L. L. & Tomei F. A. Characteristics and activities of sulfate-reducing bacteria In Sulfate-reducing bacteria (ed. Barton L. L.) Ch. 1, 1–32 (New York, 1995).

[b5] Canfield D. E. & Des Marais D. J. Aerobic sulfate reduction in microbial mats. Science 251, 1471–1473 (1991). 11538266 10.1126/science.11538266

[b6] Wang C. L., Maratukulam P. D., Lum A. M., Clark D. S. & Keasling J. D. Metabolic engineering of an aerobic sulfate reduction pathway and its application to precipitation of cadmium on the cell surface. Appl. Environ. Microbiol. 66, 4497–4502 (2000). 11010904 10.1128/aem.66.10.4497-4502.2000 PMC92330

[b7] Sigalevich P., Baev M. V., Teske A. & Cohen Y. Sulfate reduction and possible aerobic metabolism of the sulfate-reducing bacterium *Desulfovibrio oxyclinae* in a chemostat coculture with *Marinobacter* sp. strain MB under exposure to increasing oxygen concentrations. Appl. Environ. Microbiol. 66, 5013–5018 (2000). 11055957 10.1128/aem.66.11.5013-5018.2000 PMC92413

[b8] Yu H. . Microbial community functional structure in response to micro-aerobic conditions in sulfate-reducing sulfur-producing bioreactor. J. Environ. Sci. 26, 1099–1107 (2014). 10.1016/S1001-0742(13)60589-6 25079640

[b9] Lohwacharin J. & Annachhatre A. P. Biological sulfide oxidation in an airlift bioreactor. Bioresour. Technol. 101, 2114–2120 (2010). 19942429 10.1016/j.biortech.2009.10.093

[b10] Sahinkaya E., Hasar H., Kaksonen A. H. & Rittmann B. E. Performance of a sulfide-oxidizing, sulfur-producing membrane biofilm reactor treating sulfide-containing bioreactor effluent. Environ. Sci. Technol. 45, 4080–4087 (2011). 21452867 10.1021/es200140c

[b11] Celis-García L. B., González-Blanco G. & Meraz M. Removal of sulfur inorganic compounds by a biofilm of sulfate reducing and sulfide oxidizing bacteria in a down-flow fluidized bed reactor. J. Chem. Technol. Biot. 83, 260–268 (2008).

[b12] Zhang J., Dubey B. & Townsend T. Effect of moisture control and air venting on H_2_S production and leachate quality in mature C&D debris landfills. Environ. Sci. Technol. 48, 11777–11786 (2014). 25244062 10.1021/es5010957

[b13] Shen D. S. . Characteristics of H_2_S emission from aged refuse after excavation exposure. J. Environ. Manage. 154, 159–165 (2015). 25725388 10.1016/j.jenvman.2015.02.024

[b14] Fang Y. . Endogenous mitigation of H_2_S inside of the landfills. Environ. Sci. Pollut. Res. 23, 2505–2512 (2016). 10.1007/s11356-015-5482-7 26423286

[b15] Fang Y. . Sulfide oxidation and nitrate reduction for potential mitigation of H_2_S in landfills. Biodegradation 26, 115–126 (2015). 25680916 10.1007/s10532-015-9720-y

[b16] Masoud W. . Characterization of bacterial populations in Danish raw milk cheeses made with different starter cultures by denaturing gradient gel electrophoresis and pyrosequencing. Int. Dairy J. 21, 142–148 (2011).

[b17] Zhang X. Q. . The relief of microtherm inhibition for p-fluoronitrobenzene mineralization using electrical stimulation at low temperatures. Appl. Microbiol. Biotechnol. 99, 4485–4494 (2015). 25575889 10.1007/s00253-014-6357-4

[b18] Lahav O., Sagiv A. & Friedler E. A different approach for predicting H_2_S(g) emission rates in gravity sewers. Water Res. 40, 259–266 (2006). 16343590 10.1016/j.watres.2005.10.026

[b19] Zhang L. . Chemical and biological technologies for hydrogen sulfide emission control in sewer systems: a review. Water Res. 42, 1–12 (2008). 17692889 10.1016/j.watres.2007.07.013

[b20] Bernaardez L. A., Lima De Andrade L. R. P., De Jesus E. B., Ramos C. L. S. & Almeida P. F. A kinetic study on bacterial sulfate reduction. Bioproc. Biosyst. Eng. 36, 1861–1869 (2013). 10.1007/s00449-013-0960-0 23636473

[b21] Pagani I. . Complete genome sequence of *Desulfobulbus propionicus* type strain (1pr3^T^). Stand. Genomic Sci. 4, 100–110 (2011). 21475592 10.4056/sigs.1613929 PMC3072085

[b22] Rumsey I. C. & Aneja V. P. Measurement and modeling of hydrogen sulfide lagoon emissions from a swine concentrated animal feeding operation. Environ. Sci. Technol. 48, 1609–1617 (2014). 24387076 10.1021/es403716w

