# Supplementary material for: Hydrogen sulfide (H2S) emission control by aerobic sulfate reduction in landfill
Source: Sci Rep. 2016 Dec 2;6:38103. doi: 10.1038/srep38103 (PMC5133566; doi:10.1038/srep38103)
Supplement: Supplementary Information [file srep38103-s1.pdf]

SUPPORTING INFORMATION

Hydrogen sulfide (H_2S) emission control by aerobic sulfate reduction in landfill

Yuyang Long*, Yuan Fang, Dongsheng Shen, Huajun Feng*, Ting Chen

Zhejiang Provincial Key Laboratory of Solid Waste Treatment and Recycling, School of Environmental Science and Engineering, Zhejiang Gongshang University, Hangzhou, 310018, China

---

*Corresponding author.

Tel: +86 571 88832369; fax: +86 571 88832369

Email address: longyy@zjgsu.edu.cn (Y.-Y. Long); fenghuajun@mail.zjgsu.edu.cn (H.-J. Feng)

Fig. S1 Schematic diagram of simulated landfill systems (1. Leachate outlet 2. Leachate collection tank 3. Gravel layer 4. Landfill site 5.Sampling ports 6. Sandy layer 7.Headspace 8.Gas outlet 9.Leachate recirculation tank 10. Peristaltic pump 11.Leachate distribution system 12.Center vent pipe 13. Inlet)

Fig. S2 Accumulated amount of sulfate leached out from simulated landfills

Fig. S3 Accumulated amount of sulfide leached out from simulated landfills

Fig. S4 Canonical correspondence analysis of microbial communities (Samples are labeled in black; blue vectors indicate the effect of environment parameters on sample community outcomes; red taxa indicate the average CCA location of the sulfur-metabolizing bacterial genus) (P=0.001)

Table S1 The numbers of samples

Table S2 Changes of elemental sulfur content in each layer of simulated landfill (mg kg^{-1})

Table S3 Changes of thiosulfate content in each layer of simulated landfill (mg kg-1)

Table S4 The total relative abundances of SRB and SOB (%)

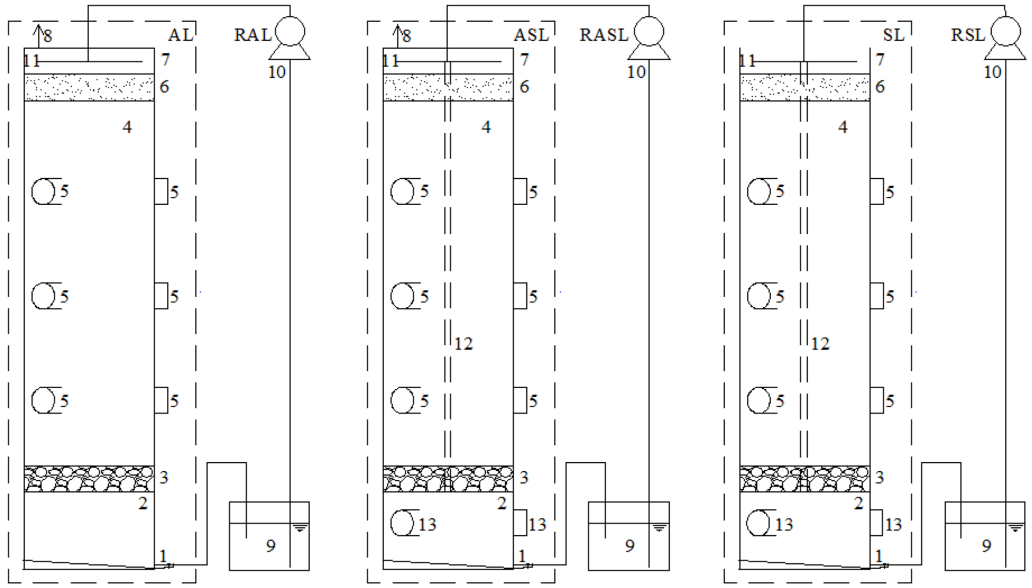

Fig. S1 Schematic diagram of simulated landfill systems (1. Leachate outlet 2. Leachate collection tank 3. Gravel layer 4. Landfill site 5.Sampling ports 6. Sandy layer 7.Headspace 8.Gas outlet 9.Leachate recirculation tank 10. Peristaltic pump 11.Leachate distribution system 12.Center vent pipe 13. Inlet)

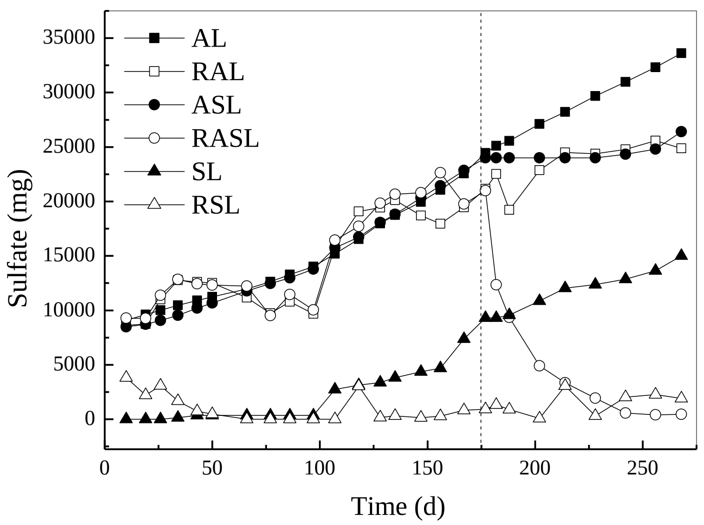

Fig. S2 Accumulated amount of sulfate leached out from simulated landfills

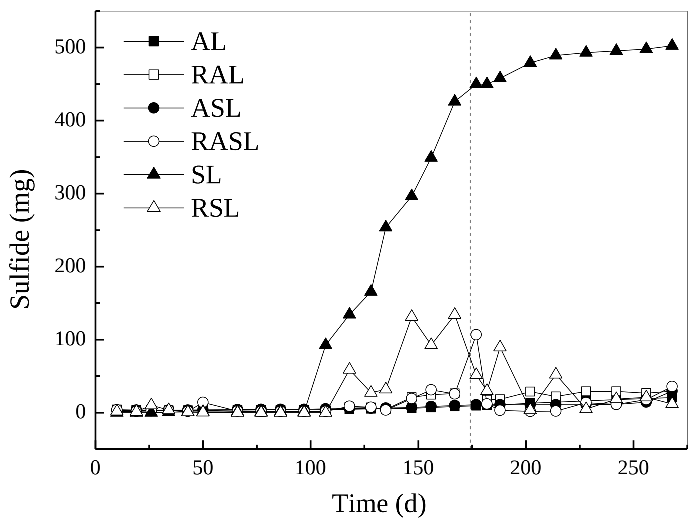

Fig. S3 Accumulated amount of sulfide leached out from simulated landfills

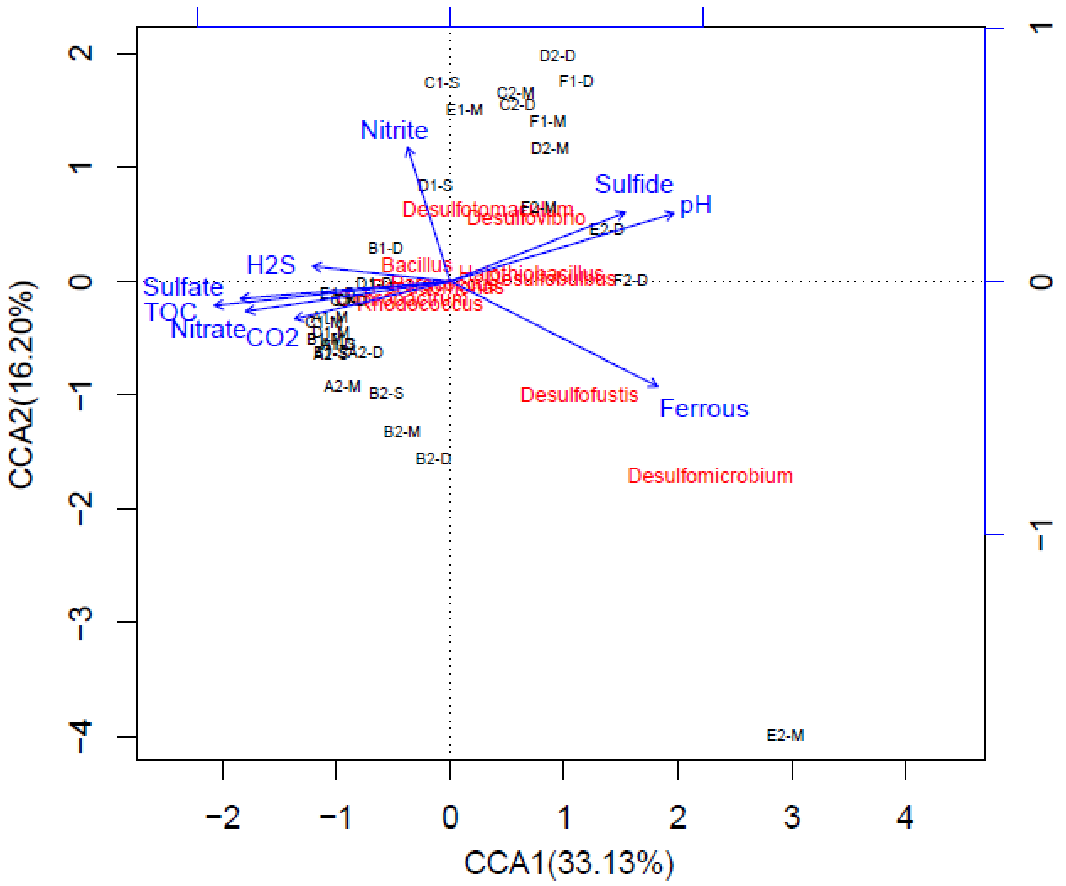

Fig. S4 Canonical correspondence analysis of microbial communities (Samples are labeled in black; blue vectors indicate the effect of environment parameters on sample community outcomes; red taxa indicate the average CCA location of the sulfur-metabolizing bacterial genus) (P=0.001)

Table S1 The numbers of samples

		0 d	191 d	269 d
AL	Shallow	CK	A1-S	A2-S
	Middle		A1-M	A2-M
	Deep		A1-D	A2-D
RAL	Shallow		B1-S	B2-S
	Middle		B1-M	B2-M
	Deep		B1-D	B2-D
ASL	Shallow		C1-S	/
	Middle		C1-M	C2-M
	Deep		C1-D	C2-D
RASL	Shallow		D1-S	/
	Middle		D1-M	D2-M
	Deep		D1-D	D2-D
SL	Shallow		/	/
	Middle		E1-M	E2-M
	Deep		E1-D	E2-D
RSL	Shallow		/	/
	Middle		F1-M	F2-M
	Deep		F1-D	F2-D

"/" indicated that the samples were not collected.

Table S2 Changes of elemental sulfur content in each layer of simulated landfill (mg kg-1)

	Time (d)	67	89	110	138	159	191	223	269
	Shallow	N.D.	N.D.	N.D.	N.D.	N.D.	N.D.	N.D.	N.D.
AL	Middle	N.D.	N.D.	N.D.	N.D.	N.D.	N.D.	N.D.	N.D.
	Deep	N.D.	N.D.	N.D.	N.D.	N.D.	N.D.	N.D.	N.D.
	Shallow	N.D.	N.D.	N.D.	N.D.	N.D.	N.D.	N.D.	N.D.
RAL	Middle	N.D.	N.D.	N.D.	N.D.	N.D.	N.D.	N.D.	N.D.
	Deep	N.D.	N.D.	N.D.	N.D.	N.D.	N.D.	N.D.	N.D.
	Shallow	N.D.	N.D.	N.D.	N.D.	N.D.	53±2	628±74	/
ASL	Middle	N.D.	N.D.	N.D.	N.D.	N.D.	18±2	30±3	437±76
	Deep	N.D.	N.D.	N.D.	N.D.	N.D.	39±3	208±5	1264±155
	Shallow	N.D.	N.D.	N.D.	N.D.	N.D.	82±5	1539±70	/
RASL	Middle	N.D.	N.D.	N.D.	N.D.	N.D.	21±4	143±16	318±3
	Deep	N.D.	N.D.	N.D.	N.D.	N.D.	61±3	352±49	1680±153
	Shallow	204±1	681±12	842±62	755±47	700±39	/	/	/
SL	Middle	N.D.	N.D.	N.D.	N.D.	N.D.	33±2	1065±88	621±63
	Deep	N.D.	N.D.	44±1	N.D.	N.D.	93±1	1568±176	1447±184
	Shallow	N.D.	N.D.	730±18	870±113	863±43	/	/	/
RSL	Middle	N.D.	N.D.	N.D.	N.D.	N.D.	N.D.	602±14	914±63
	Deep	N.D.	N.D.	N.D.	264±24	1007±54	657±19	933±40	2099±36

"/" indicated that the samples were not collected.

"N.D." indicated "not detected".

Table S3 Changes of thiosulfate content in each layer of simulated landfill (mg kg-1)

	Time (d)	89	110	138	159	191	223	269
AL	Shallow	N.D.	N.D.	N.D.	N.D.	N.D.	N.D.	N.D.
	Middle	N.D.	N.D.	N.D.	N.D.	N.D.	N.D.	N.D.
	Deep	N.D.	N.D.	N.D.	N.D.	N.D.	N.D.	N.D.
RAL	Shallow	N.D.	N.D.	N.D.	N.D.	N.D.	N.D.	N.D.
	Middle	N.D.	N.D.	N.D.	N.D.	N.D.	N.D.	N.D.
	Deep	N.D.	N.D.	N.D.	N.D.	N.D.	N.D.	N.D.
ASL	Shallow	N.D.	N.D.	N.D.	N.D.	N.D.	N.D.	/
	Middle	N.D.	N.D.	N.D.	N.D.	N.D.	N.D.	N.D.
	Deep	N.D.	N.D.	N.D.	N.D.	N.D.	N.D.	N.D.
RASL	Shallow	N.D.	N.D.	N.D.	N.D.	N.D.	N.D.	/
	Middle	N.D.	N.D.	N.D.	N.D.	N.D.	N.D.	N.D.
	Deep	N.D.	N.D.	N.D.	N.D.	N.D.	N.D.	N.D.
SL	Shallow	136±10	69±3	233±18	99±1	/	/	/
	Middle	N.D.	66±1	N.D.	N.D.	N.D.	N.D.	N.D.
	Deep	N.D.	203±9	N.D.	N.D.	N.D.	N.D.	105±6
RSL	Shallow	N.D.	204±6	301±10	145±9	/	/	/
	Middle	N.D.	N.D.	N.D.	N.D.	170±5	399±13	N.D.
	Deep	N.D.	80±2	275±2	212±1	273±9	252±2	270±40

"/" indicated that the samples were not collected.

"N.D." indicated "under detected".

Table S4 The total relative abundances of SRB and SOB (%)

		SRB			SOB		
		0 d	191 d	269 d	0 d	191 d	269 d
AL	Shallow	0.003	0.003	0.000	6.944	2.385	43.812
	Middle		0.007	0.003		5.483	48.887
	Deep		0.003	0.010		5.266	53.648
RAL	Shallow		0.003	0.010		1.528	33.195
	Middle		0.003	0.021		3.911	38.283
	Deep		0.023	0.042		2.984	42.914
ASL	Shallow		0.228	/		10.701	/
	Middle		0.002	0.154		1.679	3.169
	Deep		0.005	0.151		1.399	6.135
RASL	Shallow		0.034	/		0.404	/
	Middle		0.003	0.375		2.015	12.580
	Deep		0.014	0.355		2.260	2.515
SL	Shallow		/	/		/	/
	Middle		0.253	1.102		1.473	5.449
	Deep		0.021	0.766		5.241	5.924
RSL	Shallow		/	/		/	/
	Middle		0.251	0.198		0.225	4.147
	Deep		0.615	0.243		1.050	2.423

"/" indicated that the samples were not collected.
